# Antibody Responses to Marburg Virus in Egyptian Rousette Bats and Their Role in Protection against Infection

**DOI:** 10.3390/v10020073

**Published:** 2018-02-06

**Authors:** Nadia Storm, Petrus Jansen Van Vuren, Wanda Markotter, Janusz T. Paweska

**Affiliations:** 1 Centre for Emerging Zoonotic and Parasitic Diseases, National Institute for Communicable Diseases of the National Health Laboratory Service, Sandringham, Johannesburg 2131, South Africa; nadias@nicd.ac.za (N.S.); petrusv@nicd.ac.za (P.J.v.V.); 2 Department of Microbiology and Plant Pathology, Faculty of Natural and Agricultural Sciences, University of Pretoria, Pretoria 0002, South Africa; 3 Centre for Viral Zoonoses, Department of Medical Virology, School of Medicine, Faculty of Health Sciences, University of Pretoria, Pretoria 0001, South Africa; wanda.markotter@up.ac.za; 4 Division Virology and Communicable Diseases Surveillance, School of Pathology, University of the Witwatersrand, Johannesburg 2193, South Africa

**Keywords:** Marburg virus, Egyptian rousette bat, antibody response, maternal immunity, immune duration, reinfection, viral shedding, South Africa

## Abstract

Egyptian rousette bats (ERBs) are reservoir hosts for the Marburg virus (MARV). The immune dynamics and responses to MARV infection in ERBs are poorly understood, and limited information exists on the role of antibodies in protection of ERBs against MARV infection. Here, we determine the duration of maternal immunity to MARV in juvenile ERBs, and evaluate the duration of the antibody response to MARV in bats naturally or experimentally infected with the virus. We further explore whether antibodies in previously naturally exposed bats is fully protective against experimental reinfection with MARV. Maternal immunity was lost in juvenile ERBs by 5 months of age. Antibodies to MARV remained detectable in 67% of experimentally infected bats approximately 4 months post inoculation (p.i.), while antibodies to MARV remained present in 84% of naturally exposed bats at least 11 months after capture. Reinfection of seropositive ERBs with MARV produced an anamnestic response from day 5 p.i. Although PCR-defined viremia was present in 73.3% of reinfected ERBs, replicating virus was recovered from the serum of only one bat on day 3 p.i. The negative PCR results in the salivary glands, intestines, bladders and reproductive tracts of reinfected bats, and the apparent absence of MARV in the majority of swabs collected from these bats suggest that reinfection may only play a minor role in the transmission and maintenance of MARV amongst ERBs in nature.

## 1. Introduction

Marburg virus (MARV) is a member of the family *Filoviridae* and is the causative agent of severe and often fatal hemorrhagic fever in humans and non-human primates [1]. At least 13 outbreaks of MARV disease have been recorded to date, of which several have been associated with entry into caves or mines, or contact with bats [2,3,4,5,6,7,8,9]. The Egyptian rousette bat (ERB), *Rousettus aegyptiacus*, has been recognized as a reservoir host for MARV based on repeated isolation of the virus from naturally infected bats [10,11,12] as well as the absence of clinical disease following experimental inoculation [13,14,15,16]. Much progress has been made in identifying and studying the reservoir host for MARV in recent years [10,11,12,13,14,15,16,17,18], but knowledge of the biology of MARV within ERBs remains sparse. Spillover of MARV into human and animal populations often coincides with periods of increased viral shedding from ERBs [11], but the mechanisms driving the transmission and maintenance of MARV remain to be described. Currently, three hypotheses for MARV transmission dynamics in ERB populations exist: (1) bats may obtain lifelong immunity following recovery from a primary infection, and new outbreaks of the virus only occur when the pool of susceptible bats is replenished by weaned juveniles that have lost maternal immunity; (2) immunity to MARV in bats may be transient, with the virus being able to persist through fluctuating herd immunity; or (3) bats may be persistently infected with MARV, shedding virus periodically due to physiological or environmental stress factors [19]. Mathematical models and longitudinal ecological studies of filoviruses in ERBs have suggested that seasonal increases in viral shedding may occur in response to drivers such as waning immunity, births and migration of bats between colonies [11,20,21]. At present, the only evidence available points to the loss of maternal immunity in juvenile bats as being a major driver of MARV maintenance in nature [11], but information on exactly when juveniles lose maternal immunity and become susceptible to infection with the virus is limited.

Bat immunity is a poorly studied subject, and little is known about the role of antibody responses in the protection of ERBs against MARV infection. It also remains unclear whether primary infection with MARV results in lifelong, long term or transient protective immunity. A study by Schuh et al. recently showed that antibodies against MARV in experimentally infected ERBs declined to undetectable levels by the third month post experimental infection [16], possibly resulting in the susceptibility of these bats to MARV reinfection. Other studies have shown that antibodies may not be a major driver of viral clearance in bats [22,23], and that antibodies may even enhance MARV infection in vitro [24].

Determining the duration of actively and passively acquired immunity in ERBs, and whether actively acquired anti-MARV antibodies in ERBs are protective against reinfection, may assist in understanding how herd immunity influences MARV maintenance and population transmission dynamics. This knowledge may, in turn, assist in predicting and preventing spillover events into human and other animal populations. In this study, we show that maternal antibodies to MARV are lost in juvenile ERBs between 4 and 5 months after birth, becoming susceptible to primary infection with the virus. We also show that actively acquired antibodies to MARV in ERBs experimentally or naturally infected with the virus remain detectable in the majority of bats at 110 days post infection (67%) and 11 months after capture (84%), respectively, contrasting with the results found by Schuh et al. [16]. In a previous study, we demonstrated short term protective immunity against MARV reinfection and replication in ERBs first inoculated 48 days prior [15]. Here, we determine the role of the antibody response in the protection of previously naturally exposed ERBs against MARV reinfection. We show that antibodies do not completely protect previously MARV-exposed bats against reinfection, but effectively curbs systemic spread of the virus. The resulting lack of viral shedding implies that reinfection of previously exposed bats is not a major contributor to the transmission and maintenance dynamics of MARV in ERBs in nature.

## 2. Materials and Methods

### 2.1. Regulatory Requirements and Ethics Clearance

Egyptian rousette bats were sourced, housed and handled as previously described [13,15]. A permit for the capturing of ERBs in the Limpopo Province of South Africa (005-00002, 3-5-2012) was obtained from the Limpopo Department of Economic Development, Environment and Tourism as well as the Gauteng Department of Agriculture and Rural Development (003767, 25-5-2012). Approval for establishing an ERB breeding colony and performing experimental infections of ERBs with MARV was obtained from the Department of Agriculture, Forestry and Fisheries of South Africa (12/11/1/1/13, 4-2-2013). Ethics approval for the colonization and experimental infection of ERBs with MARV was acquired from the National Health Laboratory Service Animal Ethics Committee (AEC 136/12, 12-12-2012; AEC 139/13, 30-8-2013), as well as the University of Pretoria Animal Ethics Committee (EC056-14, 30-6-2014; H018-16, 28-11-2016).

### 2.2. Experiment 1: Duration of Maternal Immunity to Marburg Virus in Juvenile Egyptian Rousette Bats

Twenty-six juvenile ERBs were captured using harp traps and brought into captivity as previously described [13]. Upon capture, juvenile status was confirmed by observing a lack of epiphyseal-diaphyseal fusion of the long phalanges, as well as juvenile size and pelage [25]. The bats were bled on four separate occasions using previously described methods [13,15]. At the first sampling, the bats were weighed and their forearms measured using a vernier caliper (dialMax, Wiha Tools Ltd., Worcestershire, UK). The age of each bat was then estimated according to the forearm length growth curve published by Mutere [26]. In addition to wild-caught juvenile bats, 20 bats born in captivity to MARV-seropositive dams were bled and tested for the presence of maternal antibodies on five separate occasions, with sampling commencing at 3 months of age. Sera were separated from the blood samples collected from the ERBs by centrifugation at 3000× *g* for 10 min and were tested for anti-MARV immunoglobulin G (IgG) antibodies using an indirect enzyme-linked immunosorbent assay (I-ELISA). The I-ELISA was based on the recombinant MARV (Musoke) glycoprotein (GP) antigen produced in a mammalian expression system and was performed under biosafety level four conditions as previously described [15]. The negative control serum, conjugate controls and test sera were assayed in duplicate at a dilution of 1:100, and positive control serum was assayed in quadruplicate. Positive control serum was derived from a pool of serum from ERBs infected with MARV during a previous experiment [13], and negative control serum was derived from a pool of serum obtained from six MARV-naive ERBs born in captivity. Optical density (OD) values were measured at 405 nm using a microplate reader. The means of the OD values of the test sera replicates were calculated and converted to a percentage positivity (PP) relative to the positive control serum using the following equation: (mean net OD of test sera replicates/mean net OD of positive control) × 100 [27].

### 2.3. Experiment 2: Duration of the Antibody Response to Marburg Virus in Experimentally Infected Egyptian Rousette Bats

Six MARV-naive ERBs were inoculated subcutaneously with 100 µL of tissue culture supernatant containing 10^5.3^/mL tissue culture infectious dose (TCID_50_) of MARV (isolate SPU 148/99/I Watsa, second passage in Vero cells). The bats were clinically monitored and bled over a period of 110 days as previously described [13,15]. Sera were tested for anti-MARV IgG antibodies using I-ELISA as previously described [15].

### 2.4. Experiment 3: Duration of the Antibody Response to Marburg Virus in Naturally Infected Egyptian Rousette Bats

Thirty-eight bats that had previously been exposed to MARV in nature as evidenced by the presence of anti-MARV IgG antibodies in sera at the time of capture (PP value range: 22.6–176.1; cut-off value for I-ELISA: 16.8 PP) were brought into captivity as previously described [13]. The bats were monitored for the presence of anti-MARV IgG antibodies in their sera over a period of 11 months using I-ELISA as previously described [15].

### 2.5. Experiment 4: Re-Infection of Seropositive Egyptian Rousette Bats with Marburg Virus

#### 2.5.1. Inoculation of Seropositive Egyptian Rousette Bats with Marburg Virus

Seventeen wild-caught bats with MARV-specific IgG PP values ranging from 26.5 to 146.3 were selected for this experiment. Bat sera were tested for the presence of antibodies to MARV both at capture and one week prior to commencing the experiment. While the infection histories of the bats were unknown, the bats had most likely been infected with the MARV variant circulating in a cave located in the Matlapitsi Valley, Limpopo Province, South Africa [28], where the bats were captured. Fifteen bats (13 adult females and two adult males) were inoculated subcutaneously with 100 µL of tissue culture supernatant containing 10^5.3^/mL TCID_50_ of MARV/Hsap/COD/99/Watsa-SPU148-99-I, (second passage in Vero cells). Two control bats (adult females) were inoculated subcutaneously with 100 µL of Eagle’s Minimum Essential Medium (EMEM; Lonza, Basel, Switzerland). Bats were clinically monitored daily, and swabbed, bled and serially euthanized on days 0, 3, 5, 7, 9 and 12 post inoculation (p.i.) as previously described [13,15]. Results from this experiment were compared to the results obtained from naive experimentally infected bats in a previous study by our group [15].

#### 2.5.2. Real-Time Quantitative Reverse Transcription Polymerase Chain Reaction and Virus Isolation

Ribonucleic acid (RNA) was extracted from serum, swabs and the supernatant of 10% tissue homogenates in EMEM (Lonza, Basel, Switzerland) using the QIAamp Viral RNA Mini kit (QIAGEN, Hilden, Germany) according to the manufacturer’s instructions. Real-time quantitative reverse transcription polymerase chain reaction (qRT-PCR), calculation of RNA copy numbers and conversion of copy numbers to TCID_50_ equivalents in samples tested were performed as previously described [13]. Virus isolation was attempted on all specimens with qRT-PCR positive results and was performed as previously described [13].

#### 2.5.3. Virus Neutralization Index

In order to evaluate the ability of anti-MARV IgG in sera from bats naturally exposed to a local strain of MARV (see Section 2.5.1) to neutralize a genetically distinct MARV strain, a virus neutralization index test was performed. Briefly, ten-fold dilutions of MARV/Hsap/COD/99/Watsa-SPU148-99-I (second passage in Vero cells), and MARV/Hsap/ZAF/75/Ozolin (fourth passage in Vero cells), were prepared in 96 well cell culture plates (NUNC) in sextuplicate. Triplicates of each ten-fold dilution was mixed with a 1:20 dilution of pooled MARV-positive or -negative bat serum in Minimum Essential Medium (MEM) Rega-3 (Gibco, Thermo Fisher Scientific, Waltham, MA, USA) and the plate was incubated at 37 °C for 1 hour. A confluent flask of human adrenal carcinoma cells (SW-13) was trypsinized into 40 mL MEM Rega-3 containing 8% fetal calf serum (HyClone, Separations, Randburg, Gauteng, South Africa). One hundred microliters of the cell suspension was added to each well of the plate, and the plate was incubated at 37 °C in a 5% CO_2_ atmosphere for 7 days. The plate was fixed in 80% acetone (Sigma-Aldrich, St. Louis, MI, USA) and the foci stained with rabbit anti-MARV serum followed by a fluorescein isothiocyanate-labeled secondary antibody (Sigma-Aldrich, St. Louis, MI, USA). Fluorescent foci were observed using a fluorescence microscope (EVOS, Tampa, FL, USA) and the titers of the neutralized and non-neutralized viruses were determined using the method of Spearman and Karber [29].

### 2.6. Statistical Analysis

All statistical tests were performed in Microsoft Excel. Correlation between ELISA PP value and levels of equivalent viremia in reinfected bats was determined using Spearman’s Rank-Order Correlation. The Student’s *t*-test was performed to determine whether anti-MARV IgG levels differed significantly between naive [15] and seropositive MARV infected bats (two-tailed *p*-value < 0.05). The statistical significance of differences in viral load in the tissues of naive and seropositive infected bats was determined by performing the Kruskal-Wallis rank test.

## 3. Results

### 3.1. Experiment 1: Duration of Maternal Immunity to Marburg Virus in Egyptian Rousette Bats

**Figure 1:**
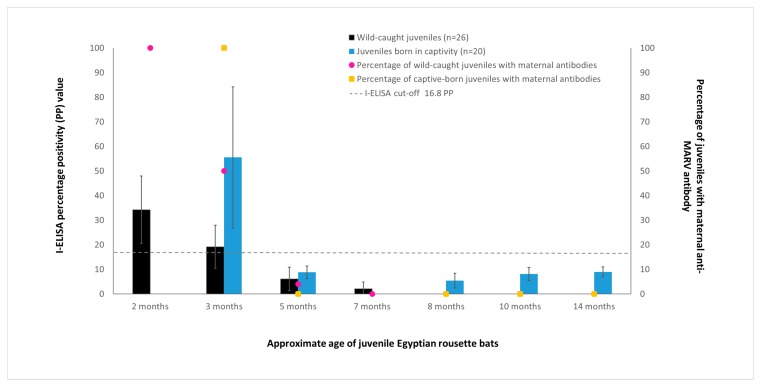
Mean maternal anti-Marburg virus (MARV) IgG antibody levels in juvenile bats born from naturally exposed mothers, with error bars representing the standard deviation of the measurements. ELISA results are shown as the percentage positivity (PP) in relation to the positive control serum. The dashed grey line represents the cut-off value of the assay at 16.8 PP (left-hand *y*-axis). The percentage of juveniles with maternal anti-MARV antibodies is displayed on the right-hand *y*-axis.

All wild-caught juveniles tested in this study were born to dams previously naturally infected with MARV as evidenced by the presence of MARV-specific maternal IgG antibodies in the juveniles (26/26) at the time of the first sampling (Figure 1). The average age of the juvenile ERBs at the first bleed was 8 weeks (range: 6–10 weeks). By the second bleed (approximately 3 months after birth), the percentage of juveniles with detectable maternal IgG antibodies to MARV had declined to 50% (13/26). At approximately 5 months after birth, maternal IgG antibodies to MARV could only be detected in a single bat, and by 7 months after birth, none of the bats had detectable levels of maternal IgG antibodies to MARV. Similarly, no maternal antibodies could be detected in juvenile bats born from captive MARV-seropositive dams at 5 and 8 months of age, even though maternal antibody titers to MARV in these bats at 3 months of age were much higher than those of wild-caught juvenile bats at a comparable age (Figure 1).

### 3.2. Experiment 2: Duration of the Antibody Response to Marburg Virus in Experimentally Infected Egyptian Rousette Bats

**Figure 2:**
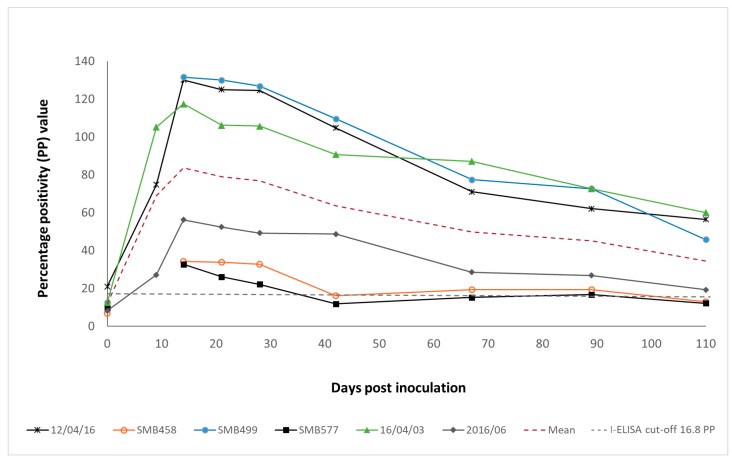
Duration of the IgG immune response to Marburg virus in individual experimentally infected Egyptian rousette bats (*n* = 6), with the dashed red line representing the mean duration of the IgG immune response. ELISA results are shown as the percentage positivity (PP) in relation to the positive control serum. The dashed grey line represents the cut-off value of the assay at 16.8 PP.

Immunoglobulin G antibodies to MARV peaked in all experimentally infected bats at day 14 p.i. (Figure 2), and then started to decline towards day 110 p.i. There was considerable variation in the immune responses of each bat, with 3 of the 6 bats producing IgG antibodies with a maximum average ELISA PP value of only 41.2. In 2 of these bats, IgG antibodies declined to undetectable levels by day 110 p.i., but IgG antibodies could still be detected in 4 of the 6 bats (67%) on this day.

### 3.3. Experiment 3: Duration of the Antibody Response to Marburg Virus in Naturally Infected Egyptian Rousette Bats

**Figure 3:**
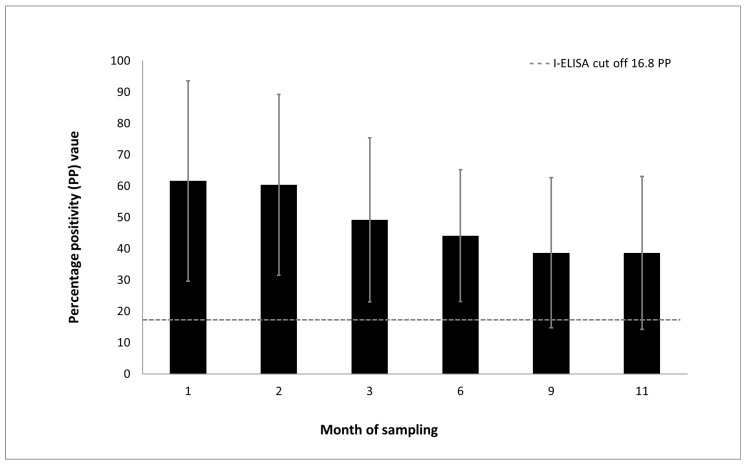
Mean duration of the IgG immune response to Marburg virus in previously naturally exposed Egyptian rousette bats (*n* = 38), with error bars representing the standard deviation of the measurements. ELISA results are shown as the percentage positivity (PP) in relation to the positive control serum. The dashed grey line represents the cut-off value of the assay at 16.8 PP.

Immunoglobulin G antibodies to MARV in previously naturally exposed bats gradually declined over a period of 11 months (Figure 3). Marburg virus-specific antibodies became undetectable in only 6 of the 38 bats (15.8%) between month 9 and 11 after capture.

### 3.4. Experiment 4: Re-Infection of Seropositive Egyptian Rousette Bats with Marburg Virus

#### 3.4.1. Serology

**Figure 4:**
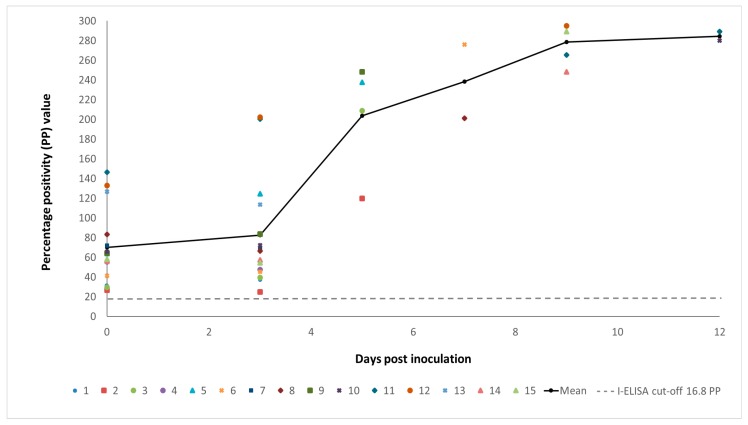
Immunoglobulin G antibody responses in 15 Egyptian rousette bats with pre-existing natural immunity following experimental infection with Marburg virus. ELISA results are shown as the percentage positivity (PP) in relation to the positive control serum. The dashed grey line represents the cut-off value of the assay at 16.8 PP.

There were no apparent signs of morbidity or mortality in any of the MARV-inoculated or control bats for the duration of the experiment. There was a statistically significant difference between the ELISA PP values of naive [15] and seropositive bats both before (two-tailed *p*-value: 0.00001), and after inoculation with MARV (two-tailed *p*-value: 0.0002). A substantial boosting effect of MARV infection on the anti-MARV GP IgG levels was noted in the MARV-inoculated bats from day 5 p.i. (Figure 4). The anti-MARV IgG levels in control bats remained unchanged for the duration of this study.

#### 3.4.2. Detection of MARV RNA by qRT-PCR and Virus Isolation

Based on qRT-PCR, 11 of the 15 seropositive bats (73.3%) were viremic on the third day p.i. (Table 1), similar to findings in naive bats infected with MARV [15]. However, the challenge virus was isolated from only one qRT-PCR positive serum (bat 4, 10^1.08^ TCID_50_/mL, ELISA PP value 47.5) on day 3 p.i. Replication of the virus in serum was also demonstrated in one additional bat (bat 2, ELISA PP value 26.5), with the level of equivalent viremia increasing from 10^0.7^ TCID_50_/mL on day 3 p.i. to 10^1.38^ TCID_50_/mL on day 5 p.i. Unlike in naive infected bats [15], MARV could not be detected in the blood of seropositive bats from day 7 p.i. Marburg virus concentrations in the serum of seropositive bats on day 3 p.i. ranged from 10^−0.09^ TCID_50_/mL to 10^2.3^ TCID_50_/mL. In comparison, MARV concentrations in the serum of naive infected bats [15] on day 3 p.i. ranged from 10^−0.3^ TCID_50_/mL to 10^2.1^ TCID_50_/mL, with no statistically significant differences between the levels of equivalent viremia in naive [15] and seropositive bats on this day (two-tailed *p*-value: 0.74).

MARV RNA was detected in the spleen of eight seropositive bats from day 3 to 12 p.i. (virus concentration range: 10^−0.6^ TCID_50_/g tissue–10^2.91^ TCID_50_/g tissue), and in the liver of six seropositive bats from day 3 to 9 p.i. (virus concentration range: 10^−0.27^ TCID_50_/g tissue–10^1.75^ TCID_50_/g tissue). In comparison, MARV concentrations in the spleens and livers of naive infected bats between days 3 and 12 p.i. ranged from 10^2.95^ TCID_50_/g tissue to 10^3.89^ TCID_50_/g tissue, and from 10^2.6^ TCID_50_/g tissue to 10^3.7^ TCID_50_/g tissue, respectively [15]. There were no significant differences between the mean MARV concentrations in the spleens (naive bats: 10^2.96^ TCID_50_/g tissue; seropositive bats: 10^2.57^ TCID_50_/g tissue; *p*-value: 0.51) and livers (naive bats: 10^1.92^ TCID_50_/g tissue; seropositive bats: 10^1.5^ TCID_50_/g tissue; *p*-value: 0.28) of naive [15] and seropositive bats on day 3 p.i. However, the virus cleared earlier in seropositive bats, with a statistically significant difference in viral loads in the livers (*p*-value: 0.01) and spleens (*p*-value: 0.02) of naive and seropositive bats from day 5 p.i.

Viral RNA was detected in the lung of one seropositive bat on day 3 p.i. (10^−0.47^ TCID_50_/g tissue) and in one nasal swab on day 5 p.i. (10^0.82^ TCID_50_/mL) (Table 1). No MARV RNA could be detected in any of the other tissues sampled in our study (Table 1). These findings differ from results obtained from a previous study of experimental MARV infection in naive bats, where MARV RNA could be detected in the salivary glands (18% of bats), kidney (9%), intestine (27%), bladder (5%) and the reproductive tract (18%) between 3 and 12 days p.i. [15]. No MARV RNA was detected in any specimens collected from control bats.

There was a negative correlation (r_s_ = −0.61, Spearman’s: *p* = 0.001) between the ELISA PP value and the level of equivalent viremia, suggesting that ERBs are to some extent more likely to become viremic upon reinfection when levels of MARV-specific IgG have declined.

#### 3.4.3. Virus Neutralization Index

The titer of MARV/Ozolin titrated on the SW-13 cells was 1.58 × 10^7^ TCID_50_/mL, and was neutralized to a titer of 1.58 × 10^6^ TCID_50_/mL by the anti-MARV/Matlapitsi antibody positive bat serum. The titer of MARV/Watsa titrated on the SW-13 cells was 3.41 × 10^6^ TCID_50_/mL and was neutralized by the bat serum to a titer of 7.34 × 10^5^ TCID_50_/mL. These results show that the bat serum was somewhat better able to neutralize the MARV/Ozolin strain, although the difference in neutralization was not statistically significant (two-tailed *p*-value: 0.47).

## 4. Discussion

While some estimates are available from previous studies [11,15], this study is the first to report on the duration of maternal immunity to MARV in both wild-caught and captive-born juvenile ERBs born to naturally MARV-exposed dams. In wild-caught juveniles, maternal IgG antibodies were present in all bats sampled at approximately 2 months of age, and were undetectable in all but one bat by 5 months of age. Likewise, in captive-born juveniles, maternal antibodies to MARV were undetectable in all bats by 5 months of age. These results show that maternal immunity is lost in juveniles between 4 and 5 months of age, making them susceptible to primary infection with MARV from 5 months of age onwards. The results are consistent with previous estimates of the duration of maternal immunity to MARV by our group [15] and are supported by the results of ecological studies of MARV in ERB populations [11,28]. A longitudinal study by Amman and colleagues [11] showed a peak in MARV infection rates in ERBs of around 6 months of age (MARV RNA detected in 12.4% of 6 month old juvenile ERBs compared to 2.7% in 3 month old ERBs and 0% in pups). In another ecological study, we were also able to demonstrate the presence of MARV RNA in three juvenile bats of at least six months of age, but not in younger juveniles or in adult bats [28]. These observations point to a protective role of maternal antibodies against MARV infection in juvenile bats. The loss of maternal immunity to MARV in juvenile ERBs therefore increases the overall susceptibility of a bat population to MARV infection and may be linked to an increased risk of viral shedding and spillover into the human population. Our results provide a possible time frame representing the period of greatest risk for human infection whilst entering caves inhabited by ERBs.

The duration of the antibody response of experimentally infected ERBs to MARV has previously been investigated by Schuh and colleagues [16]. Their results showed a peak in anti-MARV IgG antibodies at a mean of 20 days p.i. (range: 14–28 days p.i.), corresponding to the period when viral shedding becomes undetectable [16]. Immunoglobulin G antibodies to MARV then rapidly diminished and became undetectable within 3 months p.i. in both experimentally MARV-infected ERBs, and contact ERBs that had been “naturally” infected by experimentally infected ERBs [16]. In comparison, results from our current study as well as a previous study by our group [15] showed a peak in anti-MARV IgG antibody levels in experimentally infected bats at day 14 p.i. (range: 9–21 days p.i.), with antibodies still being detectable in the majority of the bats (67%) almost 4 months after infection in the current study. It is possible that some bats possess natural genetic differences that allow them to retain longer-term immunity to MARV compared to others, which may explain why some bats in our study rapidly lost immunity to MARV, while others did not.

In contrast to results obtained from experimental inoculation studies, our results in bats naturally exposed to MARV suggest that anti-MARV IgG can persist in the majority of bats at moderately high levels for longer than 11 months. However, because the exposure histories of these bats to MARV are unknown, it is possible that the longer lasting IgG immune response may have resulted from re-exposure to the virus after primary infection had already occurred.

The natural inoculation dose and ports of entry and exit of MARV in ERBs remains unclear. It was recently shown that bats may shed MARV in and possibly become infected through exposure to oral and fecal secretions [14]. Infection in this manner may result in different immune responses in ERBs than infection through sub-cutaneous inoculation, which was the inoculation route chosen for the experimental infection studies discussed in this paper [15,16]. Given the difference in results from studies of antibody-mediated immunity in experimentally and naturally infected ERBs, and possible differing routes and doses of experimental and natural MARV exposure, it is possible that results obtained in experimental infection studies might not accurately reflect what occurs upon natural exposure to MARV.

Further to evaluating the duration of the immune responses of ERBs to MARV, this study has indicated that bats harboring antibodies to MARV can become reinfected upon re-exposure with the virus, albeit with a heterologous isolate. The majority of reinfected bats in our study showed evidence of MARV in the blood, but virus presence in organs was mostly localized to the liver and spleen. This is in contrast to the systemic MARV replication observed in bats with a primary infection [15]. The presence of viremia in seropositive bats after rechallenge, and the isolation of MARV from the serum of one bat on day 3 p.i. indicates the possibility of transmission of MARV via the blood for a short period of time following reinfection.

Replicating virus could not be isolated from any of the qRT-PCR positive tissues tested in our study. Unsuccessful attempts at isolating replicating virus from qRT-PCR positive tissues could possibly be ascribed to the viral load being below the detectable level, inadequate sensitivity of the virus isolation method used, or the presence of immune complexes. It was recently shown that Ebola virus could not be isolated in Vero E6 culture from patient serum samples yielding C*t* values higher than 33.7 [30], and in an ecological study of MARV infection in ERBs in Uganda, MARV could not be isolated in Vero cells from bat tissues with C*t* values higher than 30 [11].

In this study, MARV could not be detected in major tissues that might play a role in viral shedding and transmission in bat populations, such as the salivary glands, intestine, reproductive tract and bladder [14,16]. For this reason, it appears that reinfection of previously exposed bats might not play a major role in the maintenance of MARV in natural bat populations. However, physiological or environmental stress factors such as pregnancy, social stress or poor nutrition may result in enhanced viral replication and shedding when reinfection takes place under such conditions. Furthermore, given the difference in sample size between this study (*n* = 15) and our previous study (*n* = 22) [15], it is possible that MARV RNA might have been detected in some of these tissues if a larger number of bats had been included in the current study.

The mechanism by which MARV is able to infect bats that have pre-existing immunity to the virus needs further elucidation. In a previous study, we showed that bats with laboratory induced immunity were able to efficiently control replication of the virus after re-exposure [15]. Similarly, Schuh et al. were unable to demonstrate viremia or the presence of MARV RNA in tissues sampled from MARV-reinfected bats with an apparent loss of immunity to the virus [31]. The differences in the results obtained from our previous work [15] and the current study could be attributed to the timing of challenge and the status of immunity of the ERBs during re-exposure. Unlike the study conducted by Schuh et al. in which bats had undetectable levels of antibodies to MARV prior to experimental reinfection [31], the bats used in the current study were collected with pre-existing natural immunity to MARV, without knowledge of the period that elapsed between the initial infection and reinfection in the laboratory. In addition, the rechallenge administered in both our previous work [15] and the work by Schuh et al. [31] made use of homologous virus, while the virus used in the current study was heterologous. The challenge virus used in the current study originated from a human patient from the Democratic Republic of the Congo (DRC), whereas the virus circulating in the cave in Matlapitsi Valley is genetically distinct from the DRC isolate, and closely related to the MARV/Ozolin isolate [28]. Several genetically distinct strains and variants of MARV may circulate within a single cave system [10,11,18], and it is possible that antibodies to one variant of MARV do not effectively neutralize variants that are genetically different. In our evaluation of the capability of bat serum positive for anti-MARV/Matlapitsi antibodies to neutralize MARV/Watsa, it was shown that the antibodies were better able to neutralize an isolate closely related to the Matlapitsi variant (MARV/Ozolin) than the Watsa isolate. However, the difference in the reduction of the virus titers was not statistically significant. Whether immunity to one genetic variant of MARV is fully cross-protective against infection with another in ERBs should be explored further.

The T cell responses produced following natural or experimental infection with MARV in ERBs remain to be evaluated. Antibody production does not necessarily correlate with viral clearance in bats [22,23]. In addition, neutralizing antibodies do not always confer protection in vivo, while-non-neutralizing antibodies may confer protection through mechanisms such as antibody-dependent cell-mediated cytotoxicity. The correlates of protection against MARV in ERBs need to be determined.

## 5. Conclusions

Our results show that passive immunity to MARV is lost in juvenile ERBs between 4 and 5 months of age, making them susceptible to infection with the virus and increasing the risk for spillover into the human population when these bats first become infected with MARV. Exposure to MARV resulted in IgG immune responses lasting at least 110 days p.i. in the majority of experimentally infected bats, and at least 11 months in the majority of naturally infected bats. Our results further suggest that antibodies to MARV in ERBs is likely not completely protective against reinfection. Future research should determine whether a complete loss of IgG antibodies to MARV may again lead to systemic infection and viral shedding in bats inoculated with different MARV isolates and subjected to different stress factors.

## Figures and Tables

**Table 1:** Quantitative reverse-transcription polymerase chain reaction and virus isolation results in specimens from seropositive Egyptian rousette bats experimentally inoculated with Marburg virus.

	Days after Inoculation ^a^				
	3 (*n* = 15)	5 (*n* = 3)	7 (*n* = 3)	9 (*n* = 6)	12 (*n* = 2)
Bat IDs	1, 2, 3, 4, 5, 6, 7, 8, 9, 10, 11, 12, 13, 14, 15	2, 5, 9	3, 6, 8	10, 11, 12, 13, 14, 15	11, 15
Specimen					
Serum	11/15; VI: 1/11	1/3; VI: 0/1	0/3	0/6	0/2
Rectal swab	0/15	0/3	0/3	0/6	0/2
Nasal swab	0/15	1/3; VI: 0/1	0/3	0/6	0/2
Oral swab	0/15	0/3	0/3	0/6	0/2
Vaginal swab	NS	NS	NS	0/4	0/1
Penile swab	NS	NS	NS	0/2	0/1
Liver	2/3; VI: 0/2	3/3; VI: 0/3	0/3	1/4; VI: 0/1	0/2
Spleen	3/3; VI: 0/3	2/3; VI: 0/2	1/3; VI: 0/1	1/4; VI: 0/1	1/2; VI: 0/1
Kidney	0/3	0/3	0/3	0/4	0/2
Lung	1/3; VI: 0/1	0/3	0/3	0/4	0/2
Intestine	0/3	0/3	0/3	0/4	0/2
Stomach	0/3	0/3	0/3	0/4	0/2
Rectum	0/3	0/3	0/3	0/4	0/2
Bladder	0/3	0/3	0/3	0/4	0/2
Reproductive organs	0/3	0/3	0/3	0/4	0/2
Salivary glands	0/3	0/3	0/3	0/4	0/2

Abbreviations: ID—identification number; NS—not sampled; PCR—polymerase chain reaction; VI—virus isolation. ^a^ Data represents the number of positive samples/number tested. Data designates PCR results unless otherwise stated. VI was only attempted on specimens with positive PCR results.
